# A bioinformatic analysis study of m^7^G regulator-mediated methylation modification patterns and tumor microenvironment infiltration in glioblastoma

**DOI:** 10.1186/s12885-022-09791-y

**Published:** 2022-07-04

**Authors:** Xinrui Wu, Chuanyu Li, Zhisu Wang, Yundi Zhang, Shifan Liu, Siqi Chen, Shuai Chen, Wangrui Liu, Xiaoman Liu

**Affiliations:** 1 Department of oncology and chemotherapy, Affiliated Hospital of Nantong University, Nantong, China; 2 Department of Clinical Medicine, Medical School of Nantong University, Nantong, China; 3 Department of Neurosurgery, Affiliated Hospital of Youjiang Medical University for Nationalities, Baise, China; 4 National Cancer Center/National Clinical Research Center for Cancer/Cancer Hospital, Chinese Academy of Medical Sciences and Peking Union Medical College, Beijing, China; 5 Department of Medical imaging, Medical School of Nantong University, Nantong, China; 6 Department of measurement and control technology and instruments, School of mechanical engineering, Nantong University, Nantong, China; 7 Department of Interventional Oncology, Renji Hospital, Shanghai Jiao Tong University School of Medicine, Shanghai, China

**Keywords:** m7G methylation, Immune infiltration, Signature, Biomarkers, Cancer, Health informatics, Neuroscience, Risk factors

## Abstract

**Background:**

Glioblastoma is one of the most common brain cancers in adults, and is characterized by recurrence and little curative effect. An effective treatment for glioblastoma patients remains elusive worldwide. 7-methylguanosine (m7G) is a common RNA modification, and its role in tumors has become a research hotspot.

**Methods:**

By searching for differentially expressed genes related to m7G, we generated a prognostic signature via cluster analysis and established classification criteria of high and low risk scores. The effectiveness of classification was validated using the Non-negative matrix factorization (NMF) algorithm, and repeatedly verified using training and test groups. The dimension reduction method was used to clearly show the difference and clinical significance of the data. All analyses were performed via R (version 4.1.2).

**Results:**

According to the signature that included four genes (*TMOD2*, *CACNG2*, *PLOD3*, and *TMSB10*), glioblastoma patients were divided into high and low risk score groups. The survival rates between the two groups were significantly different, and the predictive abilities for 1-, 3-, and 5-year survivals were effective. We further established a Nomogram model to further examine the signature,as well as other clinical factors, with remaining significant results. Our signature can act as an independent prognostic factor related to immune-related processes in glioblastoma.

**Conclusions:**

Our research addresses the gap in knowledge in the m7G and glioblastoma research fields. The establishment of a prognostic signature and the extended analysis of the tumor microenvironment, immune correlation, and tumor mutation burden further suggest the important role of m7G in the development and development of this disease. This work will provide support for future research.

**Supplementary Information:**

The online version contains supplementary material available at 10.1186/s12885-022-09791-y.

## Introduction

Glioblastoma is the most common adult primary brain cancer. It is the most malignant glioma of astrocytic tumors. About 50% of the primary central nervous system malignant tumors are categorized as glioblastoma, with an incidence rate of 3.2 cases per 100,000 people [1]. Despite the unremitting efforts of researchers to design new treatment strategies, primary glioblastoma has always persisted in an invasive manner and the patient mortality rates have increased accordingly [2]. Therefore, preventing the recurrence of glioblastoma is necessary to reduce its mortality. For patients with newly diagnosed glioblastoma, the standard treatment regimen consists of safe and feasible surgery, radiotherapy, and temozolomide chemotherapy for up to six treatment cycles [3, 4]. Patients with recurrence are treated by reoperation and radiotherapy. However, these methods have little effect on improving the overall survival rate [5]. Therefore, finding new and feasible treatments is a common goal among researchers.

RNA modification is a reversible and dynamically regulated process that is involved in major biological processes [6]. Transfer RNA (tRNA) modification is the most common RNA modification [7]. tRNA is a classical non-coding RNA. It functions in the translation process by providing amino acids to ribosomes according to the specific codon in the mRNA. tRNA is modified to form a precise L-shaped structure to perform these functions [8, 9]. In tRNA modification, 7-methylguanosine (m7G) is one of the most conservative modified nucleosides, which widely exists in eubacteria, eukaryotes, and a few archaea [10]. In most cases, m7G modification occurs at position 46 of the variable region [11]. m7G is installed at the 5′ cap in a co-transcriptional manner during transcription initiation. It can stabilize transcripts, prevent exonucleolysis and degradation, and regulate the mRNA life cycle [12].

For the relationship between RNA modification and glioblastoma, Cui et al. showed that mettl3 and mettl14 can inhibit tumor formation and demonstrated that m6A methylation can actually reduce the stability of key carcinogenic transcripts [13]. However, Visvanathan et al. confirmed that mettl3 is a cancer-promoting gene that can promote the survival of glioma cancer cells by stabilizing SRY box 2 (Sox2, [14]). These two contradictory experimental results make it difficult to clearly explain the effects of RNA modifications in glioblastoma. Some studies have shown that methyltransferase like 1 (mettl1) is a tumor suppressor gene in colon cancer [15]. Other reports have shown that eukaryotic translation initiation factor 4E (eIF4E) is very important for cancer cell transformation and has oncogenic potential in cancer development, as a eukaryotic translation initiation factor, it is significant to bind to the m7G cap existing at the 5 ‘- UTR of most eukaryotic mRNAs [16]. However, the relationship between m7G and glioblastoma has rarely been explored, giving us a strong curiosity in this field.

Thus, in this article, we cluster patients according to their expression of m7G-related genes and construct a scoring signature in which risk score is significantly related to clinical features and disease progression. We displayed the impact of m7G on glioblastoma as clearly as possible by using dimensionality reduction methods. Our results suggest a possible role of m7G-related genes that may indicate its potential as a therapeutic target in glioblastoma.

## Methods

### Glioblastoma dataset sources

Gene expression, clinical features, and simple nucleotide variation of glioblastoma samples were obtained from the following public databases: The Cancer Genome Atlas (TCGA), Gene Expression Omnibus (GEO), and Chinese Glioma Genome Atlas (CGGA). Specifically, GSE13041 of the GEO dataset was included in the analysis. Copy number variations (CNVs) of glioblastoma was downloaded through UCSC Xena (http://xena.ucsc.edu/). Genes related to m7G were extracted from Gene Set Enrichment Analysis (GSEA, http://www.gsea-msigdb.org/gsea/index.jsp), including GOBP_RNA_CAPPING (*n* = 34), GOMF_M7G_5_PPPN_DIPHOSPHATASE_ACTIVITY (*n* = 8), GOMF_RNA_7_METHYLGUANOSINE_CAP_BINDING (*n* = 12), and GOMF_RNA_CAP_BINDING (*n* = 19). After sorting and deleting duplicate genes, a total of 46 m7G-related and 11 immune checkpoint inhibitor (ICI) -related genes were included in the study. The specific genes were shown in Table S1. Among all the 46 obtained m7G-related genes, 44 of them were analyzed here, the reason of which was that only these 44 of 46 m7G related genes were expressed in the merge samples of TCGA, GEO and CGGA.

### Non-negative matrix factorization (NMF) clustering

NMF is a novel way of clustering. Using the NMF R package, it can extricate sample classification from difficult positions where gene space is in high dimensionality and there are too few samples to further explore [17]. With this method, 659 patient records were divided into groups A and B according to their expression levels of m7G-related genes. The classification of 659 patients into groups C1, C2, and C3 from the different genes of group A and B additionally utilized the NMF cluster method.

### Gene set variation analysis (GSVA) and enrichment analysis

GSVA is an updated algorithm to GSE, being the starting point of pathway-centric models of biology [18]. It can detect minimal changes to biological pathways and calculate pathway activity scores. In this study, we chose c2.cp.kegg.v7.4.symbols for the gene set [19], which was downloaded from the Molecular Signatures Database (MSigDB) database. The limma R package was then used to estimate the different biological processes that were enriched.

### Signature construction and nomogram formation

To verify m7G-related gene modifications in glioblastoma, the gene scoring system, named as the m7G score, was generated. After classification according to the expression levels of m7G-related genes, the differentially expressed genes (DEGs) of each group were found. Least absolute shrinkage and selection operator (LASSO) regression was cited to determine the optimal number of genes for the stability of the signature. Univariate Cox regression analysis was conducted to estimate the weight of each gene in the signature. The risk score formula is as follows:

$$Riskcore=\sum \limits_{i=1}^N\left({Coef}_i\cdot {Exp}_i\right)$$

where *Exp* is the gene expression level and *Coef* is the weight coefficient of each gene.

### Application of single-sample gene-set enrichment analysis (ssGSEA) in the tumor microenvironment (TME)

ssGSEA scoring was used to quantify the relative cell infiltration with respect to the TME in the glioblastoma patient cohort. A higher score indicates a greater abundance of cell infiltration. Specifically, the ESTIMATE score is positively related to the immune score and the stromal score, and the sum of the Estimation of STromal and Immune cells in MAlignant Tumours using Expression data (ESTIMATE) score and tumor score is unified. High tumor purity indicates the presence of more tumor cells and less immune and stromal cells within the TME [20].

### Dimensionality reduction of data

Abstract data and ambiguous display methods can impede data interpretation and comprehension. Thus, we are committed to attempting various ways of exhibition of samples. Among the emerging algorithms, the dimensionality reduction method attracted our attention. We used decision curve analysis (DCA) and principal component analysis (PCA) to better display the significance of the analysis

### Comparison of different signatures

In order to further test the practicability of our new signature, we compared it with four other GBM signatures published in recent three years [21–24], and the results were displayed by C-index and Root Mean Square (RMS) value.

### Cell culture and generation of lentiviral-transfected cell lines

The human glioma cell lines (LN18 and T98G) were obtained from the Cell Bank of Shanghai Institutes of Biological Sciences, Chinese Academy of Sciences (Shanghai, China). The LN18 and T98G cells were cultured in RPMI 1640 medium (Gibco, CA, USA) with 10% fetal bovine serum (FBS, Gibco, USA) and 1% penicillin-streptomycin solution (Gibco, CA, USA) at 37 °C in a humidified incubator containing 5% CO2. All of the cell lines tested negative for mycoplasma using a Mycoplasma Detection kit (Lonza). For generation of the inducible POLD3 knockdown cell lines was achieved using a pool of siRNA duplexe (lentiviral inducible human siRNA) using the following human PLOD3-specific siRNAs, synthesized by Genolution: #siRNA1, 5′- GGU UAA AGA AGG AAA UGG AUU − 3′; #siRNA2, 5′- GGA AGU ACA AGG AUG AUG AUG ACG ACG A - 3′. The siRNA duplexes were transfected into cells using Lipofectamine® RNAiMAX Reagent according to the manufacturer’s protocols.

### Western blot

Human gliomaLN18 and T98G cells (1 × 105) were seeded in 6-well plates. Western blot analyzes were performed as described previously [25]. Briefly, after 24 h, cells were treated for indicated times with DMSO (vehicle) or FHP01 or XAV939 (Merck). After transfected, cells were washed with cold PBS and total protein extracts obtained by adding RIPA Lysis buffer. After mechanicals detachment with cell scrapers, total lysates were collected in tubes, vortexed, and incubated for 15 min. For Western blot analysis, 10 μg of proteins derived from total lysates was loaded on 8% polyacrylamide gels with 1× of Laemmli buffer and resolved by SDS-PAGE, transferred to Immobilon-P PVDF membrane (Millipore, IPVH00010), probed with PLOD3 antibody (Themo Fisher, Product #PA5–106279) and GAPDH monoclonal antibody (Themo Fisher, Product #AM4300).

### Cell proliferation assay

The stably transfected LN18 and T98G cells were divided into negative control and siRNA1, siRNA2 transfected groups and seeded onto a 96-well plate at a density of 5 × 104 cells/ml. Next, the Cell Counting Kit-8 (CCK-8 Kit; Dojindo, Japan), based on the manufacturer’s instructions, was added to determine the proliferative capacity of cells. Optical density (OD) values were obtained at 450 nm and was measured at 1, 2, 3, 4 and 5 days after seeding using an automatic microplate reader (TEAN, Swiss). Three replicate analyses were performed for each sample.

### Transwell assay

A transwell cell migration assay was used to test the ability of cells to metastasize. The cell density of different groups was adjusted to 1 × 105 cells/ml, and 100 μl cell suspension of different groups were added to the upper chamber with or without Matrigel (Corning, USA). The medium containing 20% FBS was added in the lower 24-well plate chamber. After 24 h, the bottom LN18 and T98G cells were treated with 4% polyoxymethylene for 15 min, deionized water, and 0.1% crystal violet for 30 min. Finally, the LN18 and T98G cells migrating to the lower surface of transwell chamber were counted using a microscope in six random fields utilizing a 200x microscope.

### Statistical analysis

Chi-square or Fisher’s exact tests were used to compare categorical variables. Two groups of continuous variables were compared using a t test, while three or more groups used one-way ANOVA and Kruskal-Wallis tests. Prognostic analysis utilized the survival R package and Kaplan-Meier method to examine the difference. In the methods mentioned above, *P* < 0.05 indicated statistical significance. A receiver operating characteristic (ROC) curve was employed to validate the effectiveness of prediction. An area under curve (AUC) > 0.7 was considered prominent. R (version 4.1.2) and R Bioconductor package were the foundation of the analysis.

## Results

### Variation of m7G-related genes in glioblastoma

**Fig. 1:**
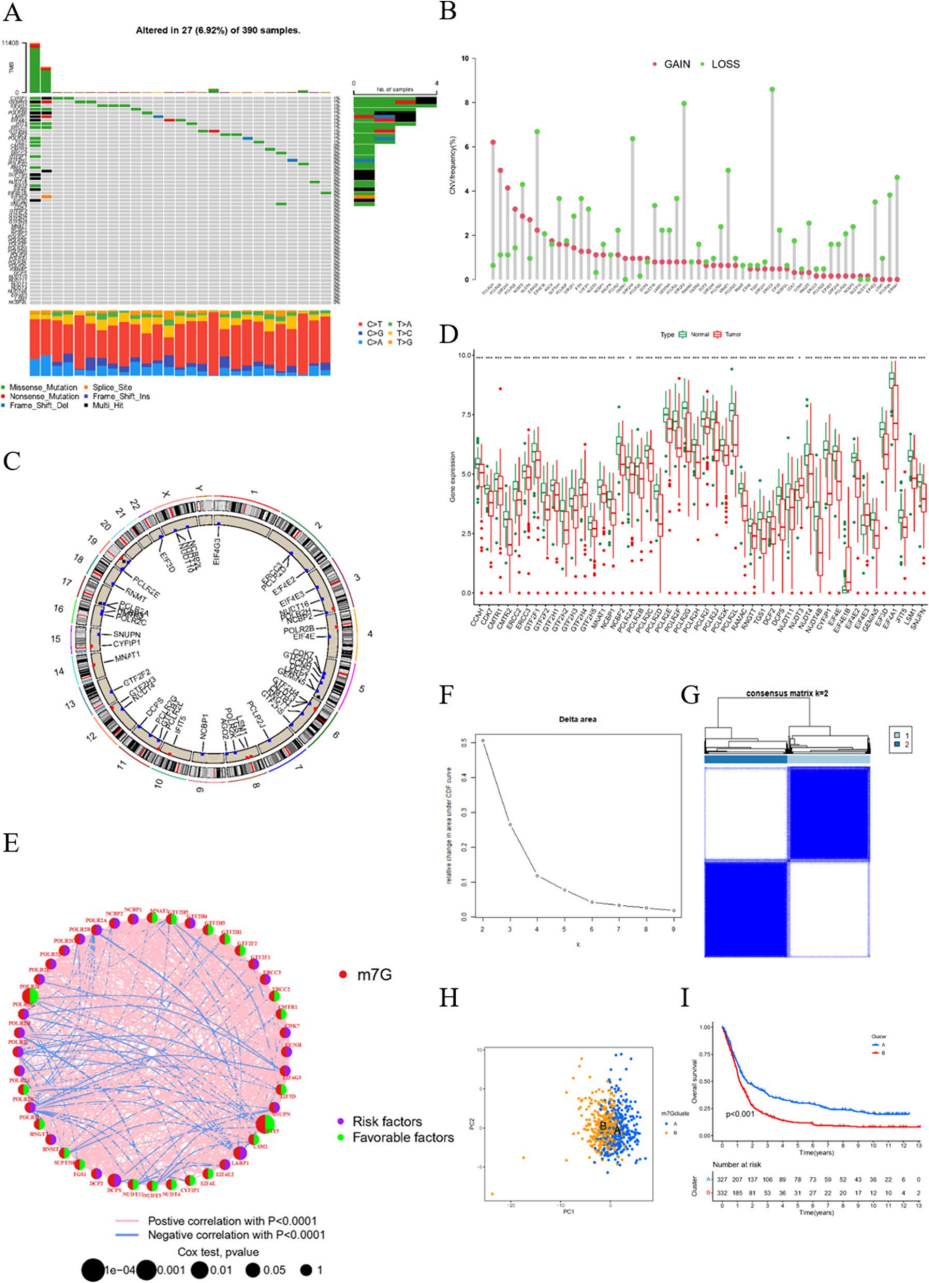
Variation of m7G related genes in glioblastoma. **A** CNV of m7G related genes in somatic cells. The mutation frequency is listed on the right. **B** Copy number of each m7G related gene in detail. GAIN infers to amplification and LOSS indicate deletion. **C** Gene location on chromosome with mutation information. Blue dots are identical to deletion and red dots are amplification. **D** Expression level of m7G related genes in normal and Glioblastoma samples. **E** Expression modification of m7G related genes and their roles in regulation. **F** Cumulative distribution function curve proves the most effect way of clustering. **G** Grouping based on the expression of m7G related genes. Group 1 indicate group A and group 2 means group B. **H** Group A and B are separated, proving the significance of grouping. **I** Survival analysis between group A and B. *P* < 0.05 is witnessed as significant

A total of 46 m7G-related genes were obtained from the GSEA dataset (Table S1). Gene mutations in somatic cells are displayed in Fig. 1A. Overall, 27 of 390 samples experienced mutations with an incidence of 6.92%. From the CNV data from TCGA cohort, the mutations of m7G-related genes in glioblastoma were displayed in detail (Fig. 1B). It turned out that the main alteration was dominated by the deletion of copy number. The relationship between gene position on the chromosome and CNV mutation was visualized through a cycle graph (Fig. 1C). Furthermore, we examined the expression levels of each m7G-related gene in glioblastoma and normal samples. The results illustrate that all m7G-related genes are distinctly expressed in normal tissues and tumors with significant differences. Interestingly, all m7G-related genes other than NUDT3, EIF4E1B, and EIF4E3 were more highly expressed in normal samples compared with tumors (Fig. 1D). The prognostic analysis of each gene was also conducted, showing highly significant different survival rates according to gene expression (Fig. S2). Consistent with the impact of genes on survival, the genes were defined as risk factors, whose expression levels were negatively correlated with survival. Favorable factors were genes with a positive relationship between expression levels and survival. The expression correlation network of m7G-related genes is depicted in Fig. 1E. The results represented above reveal the comprehensive landscape of m7G-related genes and glioblastoma.

### Clustering by m7G expression level

**Fig. 2:**
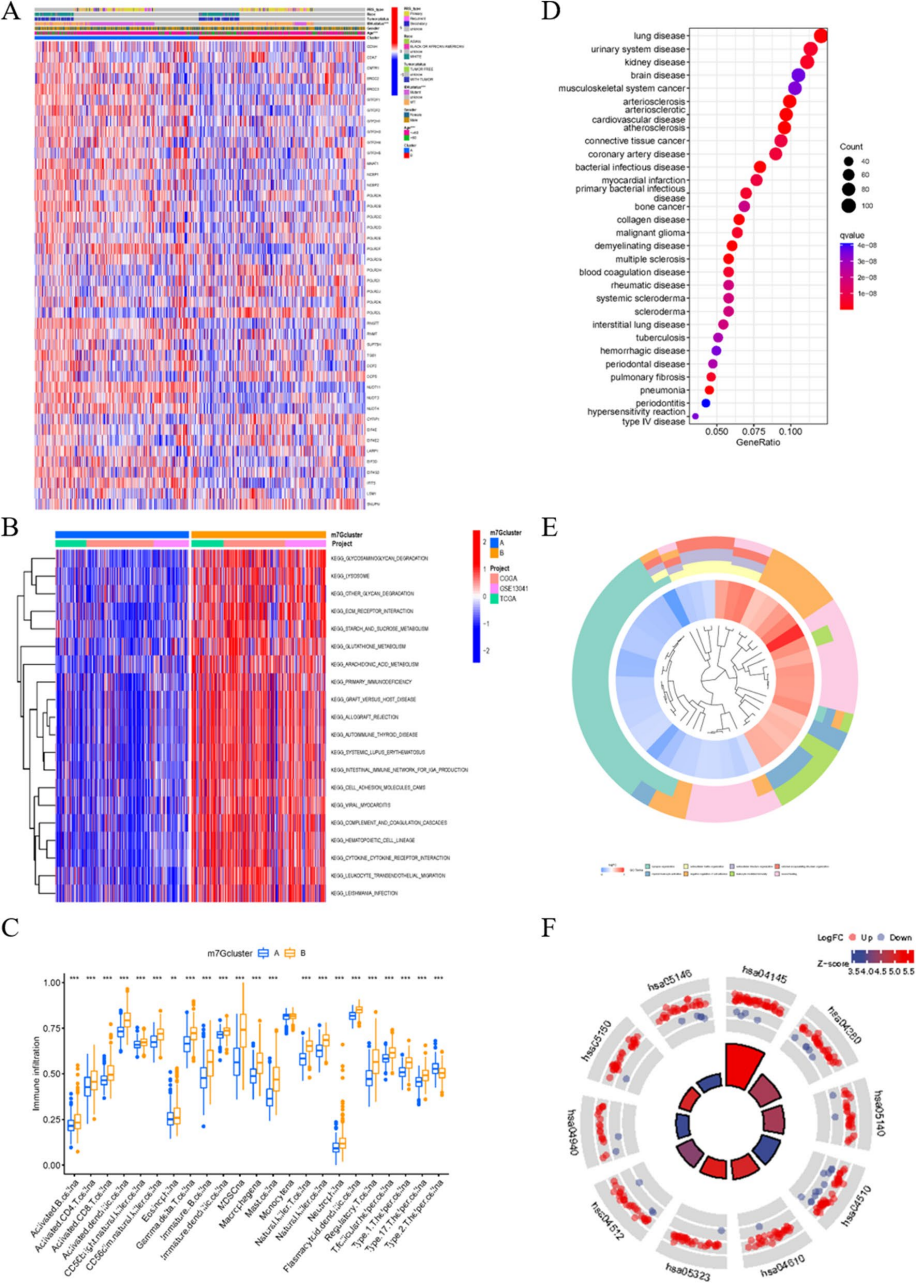
Conclusion and enrichment analysis of DEGs. **A** Heat map merging clinical information, m7G related gene expression and m7G cluster group. **B** Gene set variation analysis (GSVA) enrichment analysis of biological pathway between m7G cluster A and B. **C** Different content of immune cells in different group displayed respectively. **D** Disease Ontology analysis aiming at different expression genes (DEGs) of m7G cluster. **E**, **F** Gene Ontology and Kyoto Encyclopedia of Genes and Genomes enrichment analysis on the basis of DEGs mentioned above

With the help of the sva R package, data from TCGA, CGGA, and GSE13041 were merged to form a new sequence. When k = 2, the best clustering effect was obtained (Fig. 1F). Then, the new sequence of patients was divided into group A and B according to the expression levels of m7G-related genes (Fig. 1G). The PCA dimensionality reduction method validated the effectiveness of the grouping (Fig. 1H). The survival status is clearly significantly different between group A and group B (Fig. 1I). The relationship between the grouping, data source, and clinical manifestation is depicted in Fig. 2A. GSVA pathway enrichment analysis according to grouping shows that pathways in group A are downregulated, while upregulated in group B (Fig. 2B). The allocation is also significantly related to immune cells, implying an interaction between m7G and immune-related processes (Fig. 2C). To examine the genome wide changes between the two groups, 1253 genes with a prominent expression difference were listed (Table S2). Notably, the different genes are related to brain disease in accordance with disease ontology analysis (Fig. 2D and Table S3). Gene ontology (GO) enrichment analysis indicates a negative correlation between genes obtained above and synapse organization, and a positive correlation of leukocyte-mediated immunity and myeloid leukocyte activation (Fig. 2E and Table S4). Kyoto Encyclopedia of Genes and Genomes (KEGG) enrichment analysis shows an accumulation of genes in synapse-related pathways, such as the dopaminergic synapse and synaptic vesicle cycle, and tumorigenesis, such as transcriptional misregulation in cancer and glioma (Fig. 2F and Table S5). These results suggest that m7G participates in the development of glioblastoma through synapse-related pathways.

**Fig. 3:**
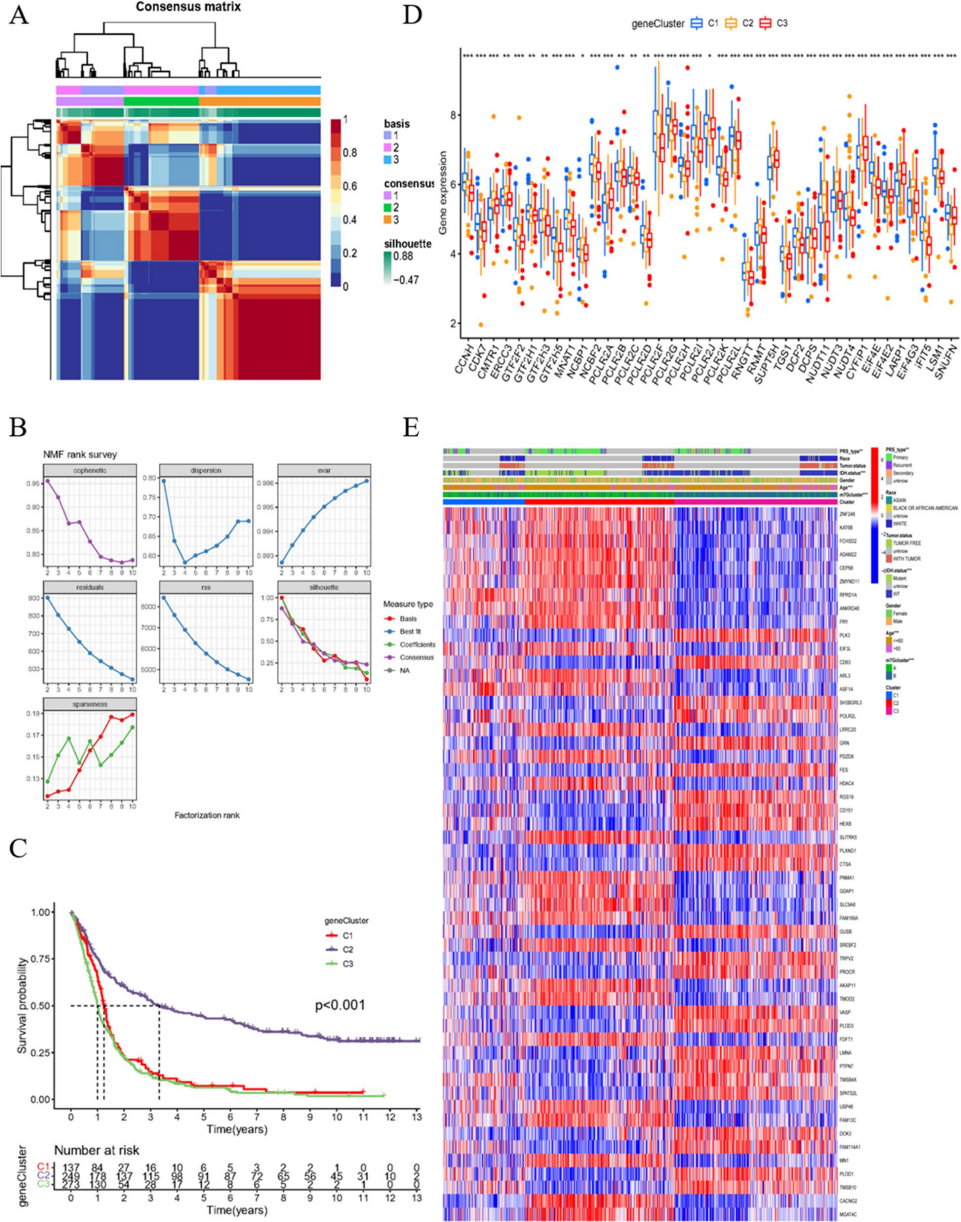
Gene cluster of glioblastoma patients. **A** Non-negative matrix factorization (NMF) algorithm confirm that three group is the most suitable way of classification. **B** The effectiveness of different grouping methods according to NMF algorithm. **C** Survival analysis of different gene clusters with *p* < 0.05 as statistically significant borderline. **D** Expression of m7G related genes in gene clusters C1, C2 and C3. **E** Heat map of clinical information, clusters and DEGs

Consistent with the DEGs, patients were grouped into three portions with the best effect, demonstrated by the NMF algorithm (Fig. 3A, B). The survival time and expression levels of m7G-related genes are remarkably different among these groups, demonstrating the conspicuous diversity and effectiveness of the division (Fig. 3C, D). The different gene expression levels of each patient and clinical features were distributed by group (Fig. 3E). Most of the genes had opposite expression patterns between groups C2 and C3, while group C1 showed random expression patterns.

### Foundation of the m7G score signature

**Fig. 4:**
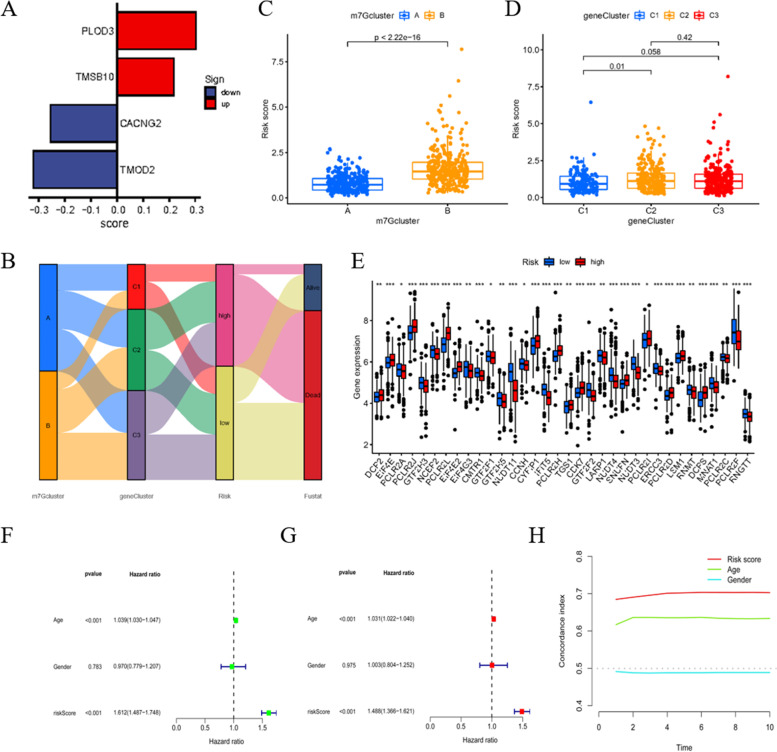
Construction of signature and statistically validation. **A** Column chart represents the weight of genes in the signature. **B** Sankey diagram to better state the modeling progress. **C**, **D** Phenotypic relationship between cluster and risk score. **E** Relationship of m7G related genes in high and low risk score group. **F**, **G** Forest maps illustrate uni and multi variant cox regression analysis. HR > 1 demonstrates that the element is risk factor. **H** C-index value estimates the probability that the predicted results are consistent with the actually observed results. In this diagram, our signature has the best performance

To further investigate the predictive value of these genes, we aimed to establish a prognostic signature for analog computation. First, by merging the expression levels of the different genes and survival information, 53 prognosis-related differential genes were identified through univariate Cox regression with hazard ratio (HR) < 0.6 and HR > 1.6 as critical values (Table S6). Then, the merged group of patients was separated to the training group (*n* = 330) and test group (*n* = 329) with the assistance of the caret R package. Next, using the data from the training group, four genes (TMOD2, CACNG2, PLOD3 and TMSB10) were selected to form the signature. The risk score calculation formula is as follows: risk score = (− 0.3206 × *TMOD2* expression) + (− 0.2556 × *CACNG2* expression) + (0.3019 × *PLOD3* expression) + (0.2177 × *TMSB10* expression) (Fig. 4A). Patients in the training group were marked as high risk or low risk relative to the median risk score value (1.1168). Patients in the test group and the entire samples were also considered as high- or low- risk group using the median risk score. The modeling process is displayed in Fig. 4B.

After calculating the risk score of each patient, groups A and B have notably different risk scores, while the differences among C1, C2, and C3 are not that prominent (Fig. 4C, D). The low and high risk score groups have remarkably different m7G-related gene expression levels (Fig. 4E). Univariate Cox regression indicated that age and risk score are relevant to prognosis (Fig. 4F). Multivariate Cox regression further confirmed that these three elements are independent prognostic factors (Fig. 4G). The concordance index comparison of each element illustrates that our signature has the best predictive accuracy compared with age and gender (Fig. 4H). These results initially verify the accuracy and feasibility of the signature.

### Validate of the signature at the clinical level

**Fig. 5:**
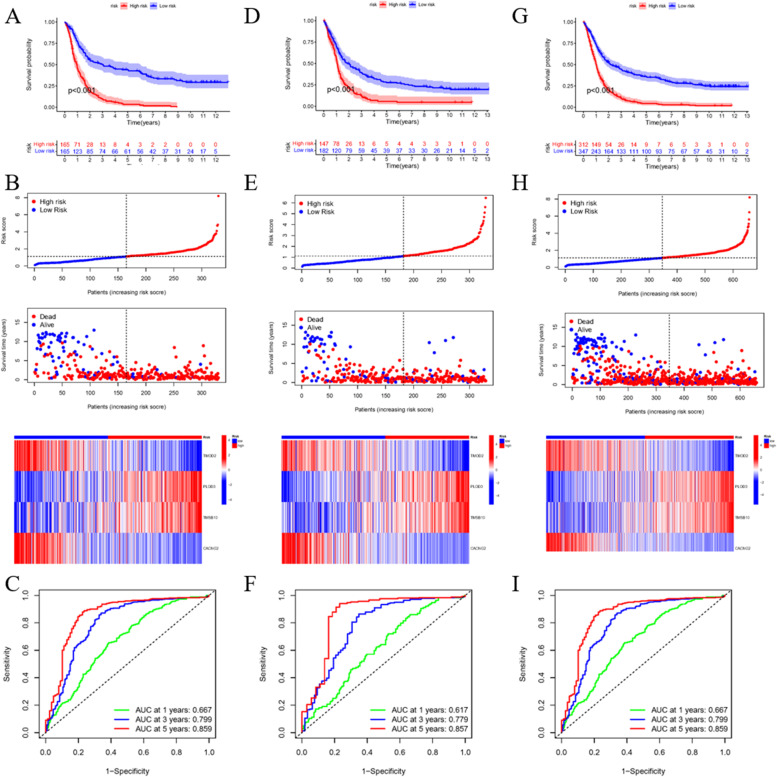
Signature consolidation through clinical information. **A** Survival analysis of high and low risk score group in train session. **B** Risk curve, survival status and gene expression of each patient in train session. **C** Receiver operating characteristic (ROC) curve in order to testify the prediction ability. Area under curve (AUC) > 0.7 is considered as ideal state. **D-F** Same analysis in test session. **G-I** Same analysis utilizing all patients’ information

To further identify the applicability of the signature in clinical characteristics, we tested and verified our signature using clinical indexes. First, we performed survival analysis between the high and low risk score groups with respect to gender, age, isocitrate dehydrogenase (IDH) status, RPS type, race, and tumor status using all patient data. The results confirm that patients with different gender, age, IDH status, and RPS type have varied survival times, while race and tumor status do not influence survival (Fig. S2). Next, in the training group, the survival differences between the high and low risk score groups are significant (Fig. 5A). Additionally, patients with low risk scores are clearly more likely to survive and have higher expression levels of *TMOD2* and *CACNG2*, but lower expression levels of *PLOD3* and *TMSB10* (Fig. 5B)*.* These results are consistent with the former verification and risk score algorithm. The ROC curve demonstrated the predictive ability (Fig. 5C) of this approach. The same analysis was conducted in the test group (Fig. 5D-F) and the whole dataset (Fig. 5G-I), and the results were consistent with those of the training group.

**Fig. 6:**
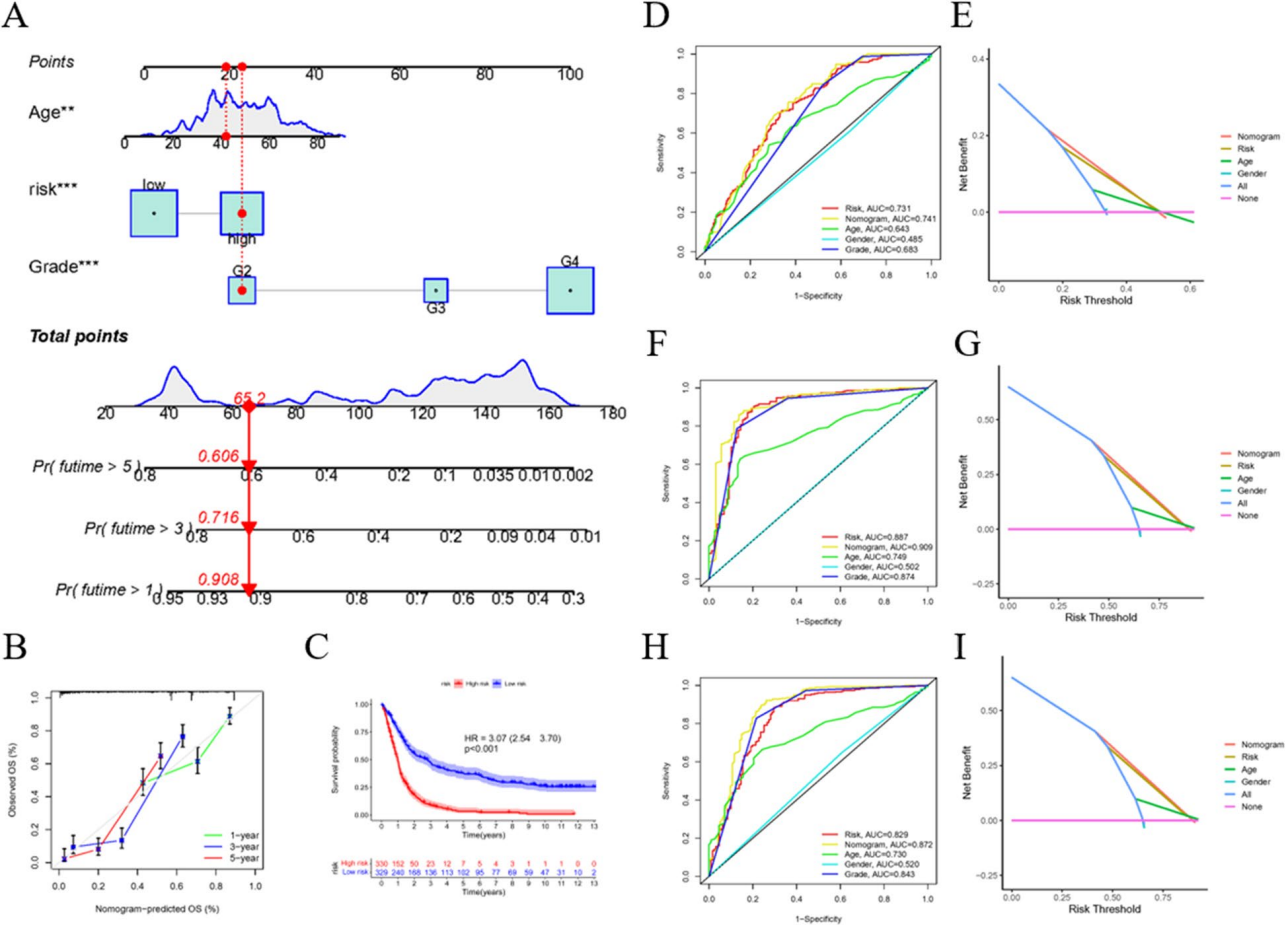
Establishment of nomogram and its forecast performance. **A** A putative displaying pattern of influence factors and weight of clinical model. **B** Calibration curves of nomogram to ascertain the prediction. **C** Overall survival of Nomogram model. **D** Receiver operating characteristic (ROC) curve shows the 1-year forecast probability through m7G score signature, nomogram, age and gender separately in detail. **E** Decision curve analysis (DCA) dimensionality reduction method to illustrate the accuracy of forecast. **F**, **G** Same analysis in 3-years prediction. **H**, **I** Same analysis in 5-years prediction

After validating the m7G signature, another forecast pattern reconciling the risk score and clinical features, such as age, was generated. The nomogram vividly displays the weight of each factor and prognostic indication of the different scores (Fig. 6A). The calibration curve indicates the unity of the observed and predicted 1-, 3-, and 5-year survival rates (Fig. 6B). According to 659 GBM samples, we divided the new risk value of the Nomogram into high and low groups, as is shown in Fig. 6C. Compared with the low-risk group (*n* = 329), the OS of the high-risk group (*n* = 330) was significantly lower, in which HR = 3.07 (2.54–3.70), *p* < 0.001. The ROC curve of 1-year prediction shows that the AUC of the risk signature, nomogram model, and age are all > 0.6, representing the effectiveness of prediction (Fig. 6D). Using the DCA dimension reduction method, the predictive efficiency is well exhibited (Fig. 6E). The same analysis was performed to analyze the 3-year (Fig. 6F, G) and 5-year (Fig. 6H, I) prediction results. All of the results described above show the effectiveness of the nomogram model.

### Broad relevance to immune cells, TME, tumor mutational burden (TMB), and stem cells

**Fig. 7:**
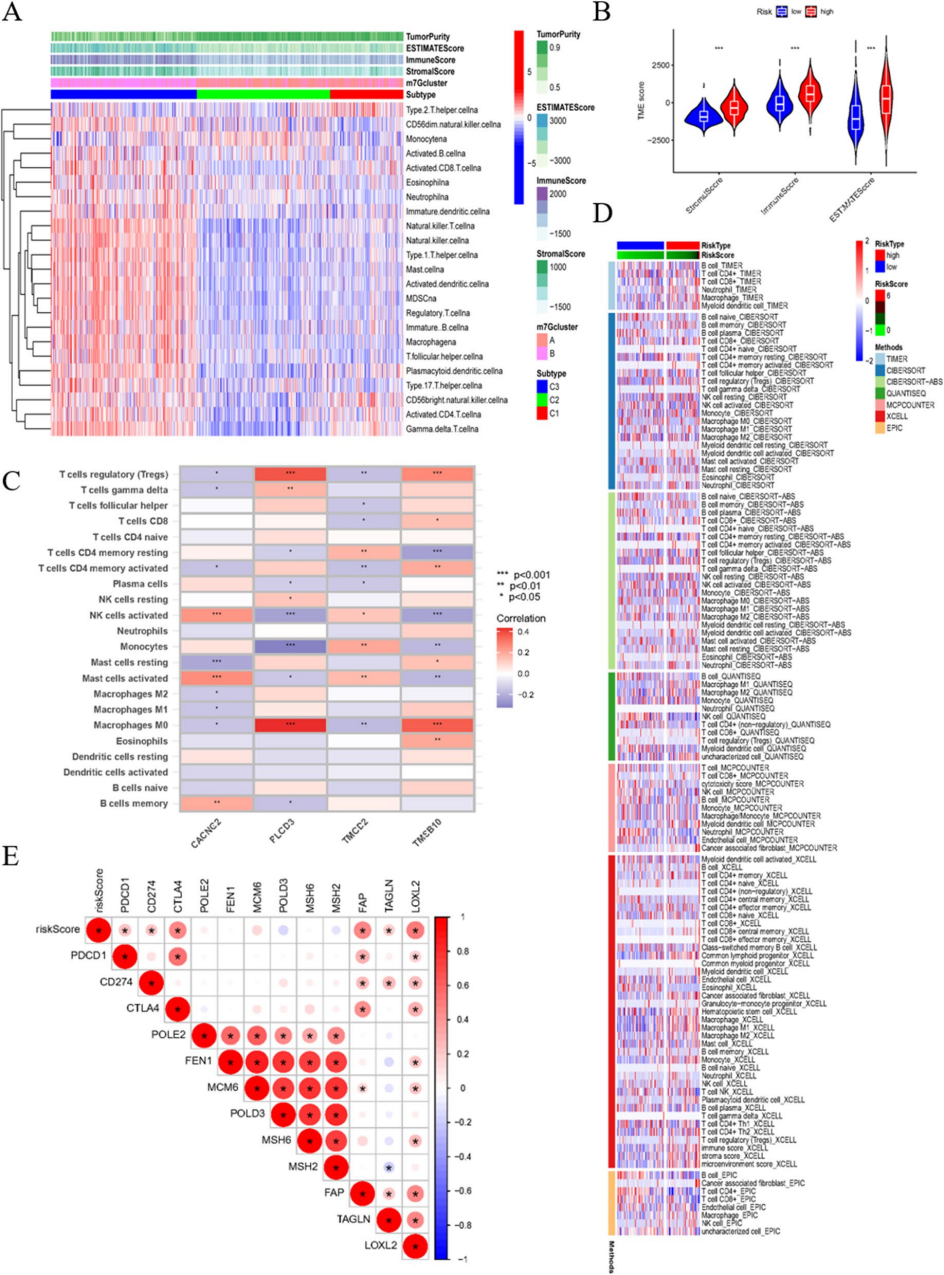
The relationship of signature and TME. **A** Single-sample gene-set enrichment analysis (ssGSEA) measured tumor miccroenvironment (TME) score and content of immune cells in each sample. **B** TME score in high and low immune group displayed comparatively. **C** Relevance of genes and immune cells using cibersort algorithm. **D** The relationship of immune cells and high and low risk score through 7 different algorithms. **E** The expression relationship of risk score and immune checkpoint inhibitor (ICI)

From the former analysis, we became interested in the relationship between m7G and the TME. The heat map displays that group C3 has higher stromal scores and immune scores, indicating that group C3 has an abundance of stromal and immune cells. There are relatively fewer tumor cells present, thus resulting in lower tumor purity scores (Fig. 7A). The definite value is demonstrated in Fig. 7B. Furthermore, we explored the intersection of immune cells. The correlations between the genes of the signature and immune cells were analyzed using the cibersort algorithm and is displayed in a heat map (Fig. 7C). The relevance of the risk score and immune cells via different calculation methods is exhibited in Fig. 7D. The risk score is positively related to immune checkpoint inhibitor genes, most notable of which are *PDCD1* (*PD1*) and *CD274* (*PD-L1*) (Fig. 7E).

**Fig. 8:**
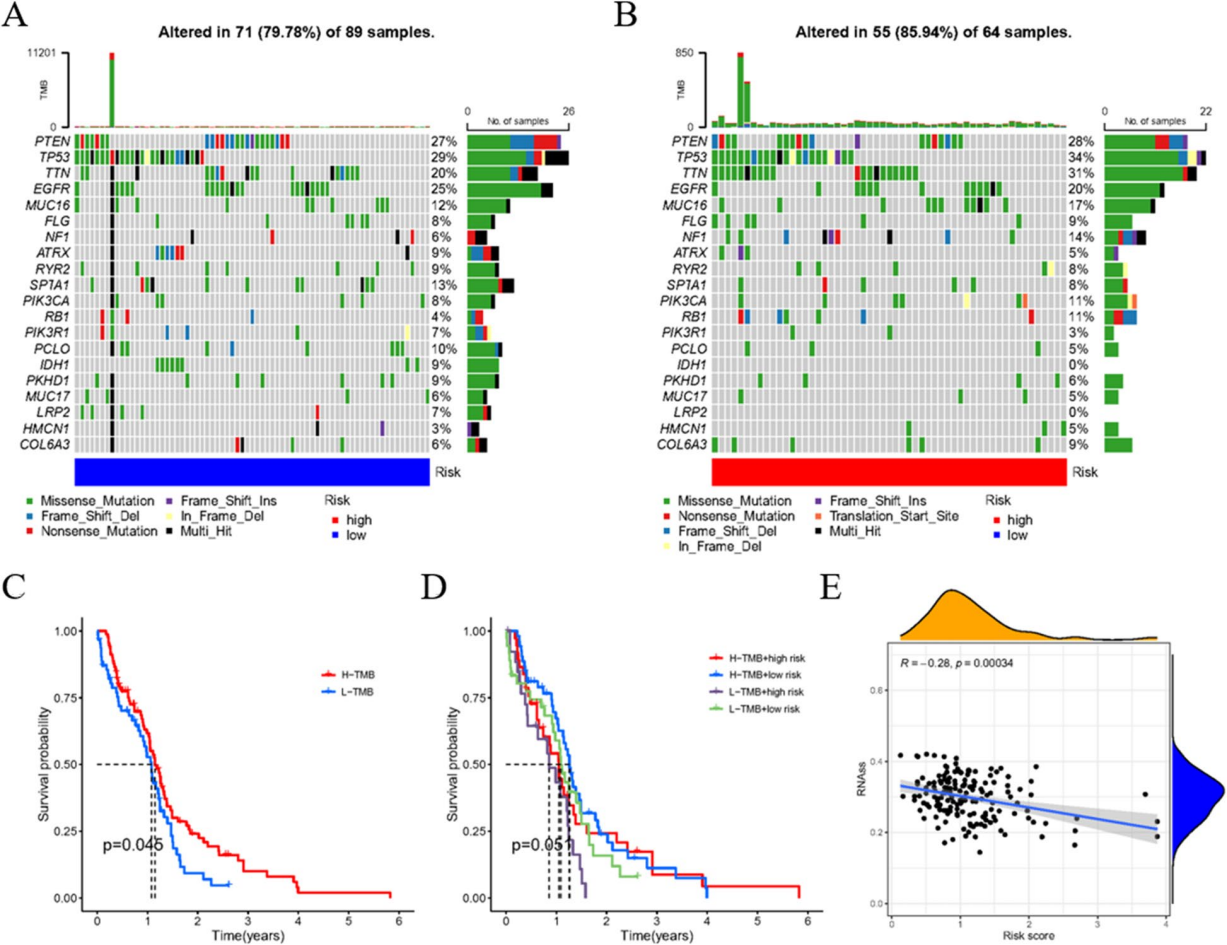
The influence of risk score clustering on TMB. **A**, **B** TMB in low and high risk score group. **C** Survival forecast analysis compared high-TMB group with low-TMB group. **D** Merging comparison of risk score group and TMB group. **(E)** Relevance of steam cell index and risk score of the signature

We also investigated the relationship between the tumor mutational burden (TMB) and risk score. The results indicate that the low risk score group has lower mutation percentages in many genes, especially *TP53*, *TTN*, and *NF1*, with 5, 11, and 8% respective decreases compared with the high risk score group (Fig. 8A, B). Then, patients were divided into high mutation and low mutation groups. Survival analysis suggested a meaningful difference between these two groups (Fig. 8C). However, when mutation risks were reconciled with risk score, the differences of survival became vague (Fig. 8D). In addition, we determined that the stem cell index is negatively related to risk score (Fig. 8E), suggesting a correlation between glioblastoma stem cells (GSCs) and m7G, and implying a research direction for future in-depth investigation.

### Comparison of m7G and other GBM related sinatures

**Fig. 9:**
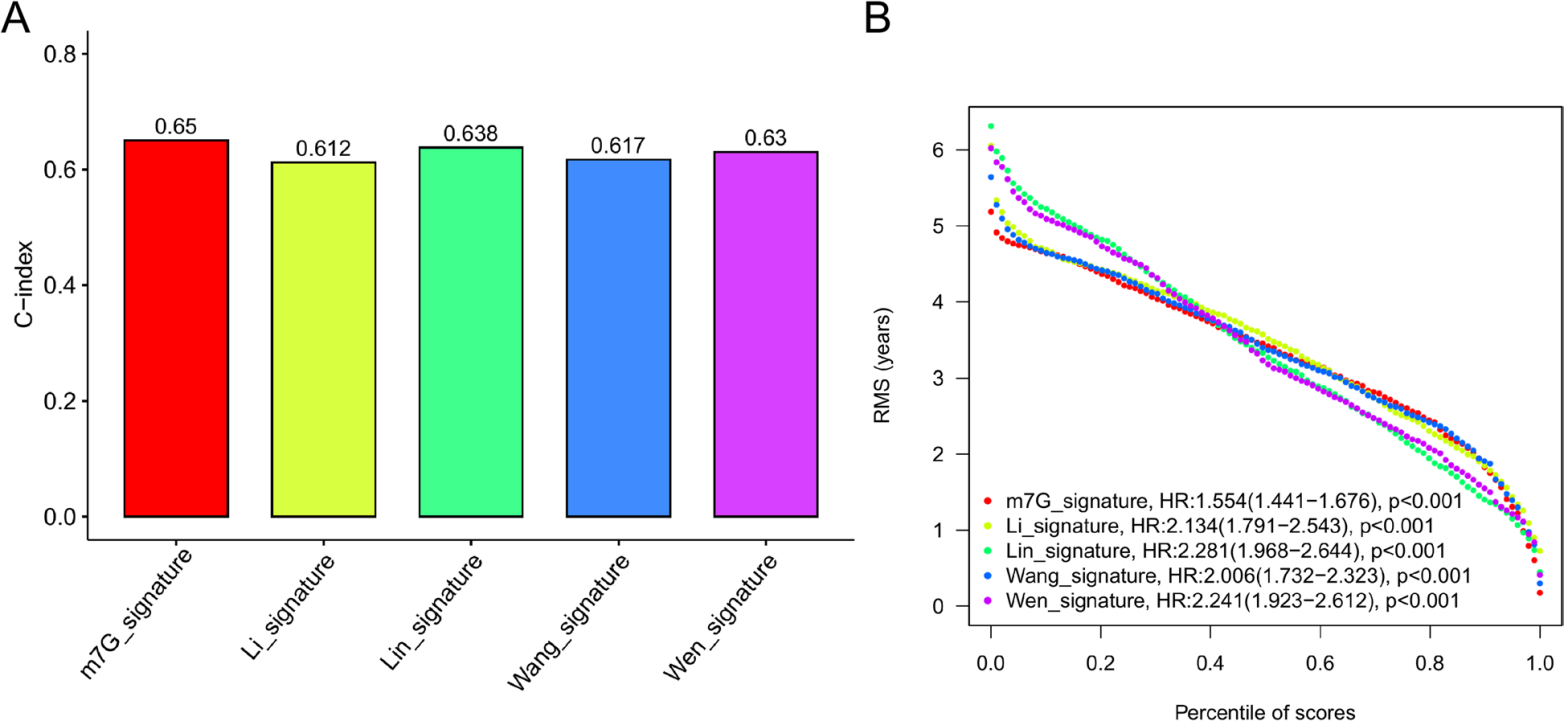
Signature comparison. **A** Comparison of the C-index value between m7G and four individual signatures. **B** Root Mean Square (RMS) values among five signatures

Four signatures about GBM which were published within three years were selected to compare to our ones. The results were shown in Fig. 9A, B. The C-index value of the signature of GBM related genes in the manuscript is the highest, which is 0.65, also, the RMS value is the smallest (HR: 1.554, *p* < 0.001), representing low dispersion and high reliability.

### Down-regulation of PLOD3 restrained proliferation and migration abilities of glioma cells

**Fig. 10:**
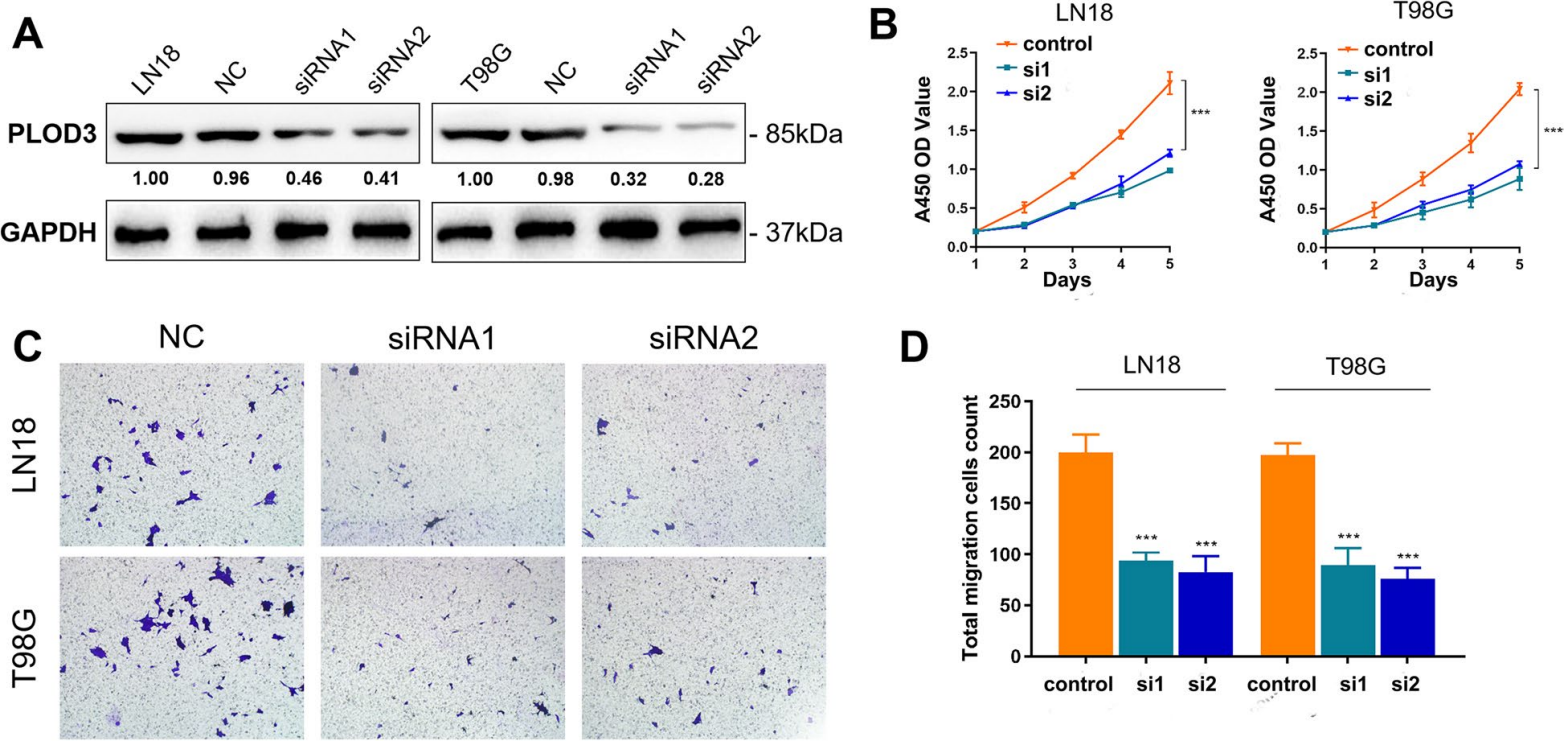
Experimental verification of PLOD3. **A** Biological behaviors of down-regulated expression of PLOD3 in LN18 and T98G cells. **B** Results of the CCK-8 assay of the influence of the PLOD3 expression on glioma cells. **C**, **D** Outcomes of the Transwell cell migration assay of PLOD3

To reveal malignant behaviors of the hub gene modulating m7G modification patterns of glioma, we first validation biological behaviors of down-regulated expression of hub gene, PLOD3, in LN18 and T98G cells (Fig. 10A, S3, S4). According to the results of the CCK-8 assay, down-regulated level of PLOD3 expression significantly restrained the proliferation ability of glioma cells compared with control group (Fig. 10B). Transwell cell migration assay also indicated that the down-regulation level of PLOD3 expression significantly inhibited the metastasis ability of glioma cells compared with control group (Fig. 10C, D). Overall, the down-regulation of PLOD3, the hub gene modulating m7G modification patterns, significantly suppressed proliferation and migration capacity of glioma cells.

## Discussion

Although emerging evidence has demonstrated the potential role of m7G in cancer and tumorigenesis, research on m7G in cancer is still relatively insufficient. In this study, we first displayed the overall mutation profile of m7G-related genes in glioblastoma samples using array data from the TCGA, CGGA, and GEO public datasets. Notably, a novel detection method called m7G Mutational Profiling sequencing (m7G-MaP-seq) has already been discovered to detect internal m7G modifications [26]. Although the methodology is different, the purpose of this technique is also to further investigate the role of mutation modification patterns in disease progression. This emphasizes the goal of our research from another perspective. The grouping process is guided by the NMF algorithm, indicating the most meaningful and distinctive cluster method. The construction of a prognostic signature showed remarkable significance and can act as an independent prognostic factor with a strong predictive effect. The cut off value of the risk score was 1.1168, and proved to be significant, as the survival analysis, TMB, and TME analyses were all statistically different.

Glioblastoma is a focus of many researchers because of its high recurrence rate and poor treatment effects. According to the WHO classification standard updated in 2016, which is based on the mutation status of the IDH 1 or 2 genes, wild-type genotypes accounted for more than 90% of cases [27]. This typing method has strong prognostic significance in this disease. For treatment methods, Han [28] summarized new molecular therapies that target mutant IDH, while therapies for wild-type genotypes need additional research. m7G has recently become a research hotspot, with many researchers becoming particularly interested in its role in tumorigenesis. m7G is a form of RNA methylation, in addition to N6-methyladenosine (m6A), 2-O-dimethyladenosine (m6Am), N1-methyladenosine (m1A), and 5-methylcytosine (m5C) [29]. Numerous studies have confirmed that m7G cap is a unique molecular module that can recruit cellular proteins and mediates cap-related biological functions, such as pre-mRNA processing, nuclear output, and cap-dependent protein synthesis [30]. Some articles have shown that RNA modifications are involved in cancer development and progression, including one by Barbieri [31]. However, the specific role of m7G in tumorigenesis is remains understudied. Therefore, we conducted a detailed analysis on the role of m7G in glioblastoma to address this gap in knowledge.

The scoring system composed of a m7G prognostic signature and grouping boundary value proved to be effective in many aspects. *Tmod2* regulates the stability of F-actin and dendrite developing during dendrization and synaptic formation. These findings provide new insights into the actin regulatory mechanisms of neuronal development, revealing potential pathways that are disrupted in many neurological disorders [32]. *CACNG2* can affect the susceptibility to postoperative chronic pain [33] or chronic pain caused by nerve injury [34]. Existing research shows that elevated expression levels of *PLOD3* can accelerate tumor progression and indicate poor prognosis [35], which is consistent with our signature where the weight of *PLOD3* was positive. Although the role of *TMSB10* in glioblastoma has not been studied, existing publications have shown that *TMSB10*, as a cancer-promoting gene, can promote cell invasion and cancer progression. This has been demonstrated in gastric cancer [36], renal clear cell carcinoma [37], and lung cancer [38]. The abovementioned results support the complex relationship between these fours genes and cancer, which is quantified by the risk score calculation formula in glioblastoma. Our correlation analysis of immunity lays a foundation for the exploration of immunotherapy in glioblastoma. Subsequent analysis also verified the relationship between m7G and the TME and TMB. Glioblastoma shows significant cellular heterogeneity, among which stem-like GSCs was the most significant [39]. There is increasing evidence that GSCs play an important role in tumor growth and treatment responses [40]. Therefore, we studied the correlation between glioblastoma and stem cells.

Based on the public database, four novel genes were discovered that are likely to be related to m7G. They are likely to affect the development and prognosis of GBM through corresponding pathways. We selected PLOD3 with the largest risk coefficient for experimental verification. As we guessed, the down-regulation of PLOD3 significantly inhibited the proliferation and migration of glioma cells. Our research strongly addresses the current gap in the m7G and glioblastoma research fields, utilizes a macroanalysis of the phenotypes of m7G-related genes in glioblastoma, establishes a prognosis evaluation system, and quantifies the impact of m7G on glioblastoma at the micro level. Overall, our work lays a solid foundation for future research.

## Supplementary Information

**Additional file 1.**

**Additional file 2.**

**Additional file 3.**

**Additional file 4.**

**Additional file 5.**

**Additional file 6.**

**Additional file 7.**

**Additional file 8.**

**Additional file 9.**

**Additional file 10.**

## Data Availability

The data sets provided in this study could be found in the online repository. The names and login number(s) of the repositories can be found in the article. https://portal.gdc.cancer.gov/;https://www.ncbi.nlm.nih.gov/geo/;http://www.cgga.org.cn/.

## Abbreviations

M7G: N7-methylguanosine
NMF: Non-negative matrix factorization
M6A: N6-methyladenosine
TCGA: The Cancer Genome Atlas
GEO: Gene Expression Omnibus
CGGA: Chinese Glioma Genome Atlas
GSEA: Gene Set Enrichment Analysis
GSVA: Gene set variation analysis
DEGs: Different expression genes
eIF4E: Eukaryotic translation initiation factor 4E
ICI: immune checkpoint inhibitor
MSigDB: Molecular Signatures Database
ESTIMATE: Estimation of STromal and Immune cells in MAlignant Tumours using Expression data
NUDT3: Nudix hydrolase 3
EIF4E1B: Eukaryotic translation initiation factor 4E family member 1B
EIF4E3: Eukaryotic translation initiation factor 4E family member 3
LASSO: Least absolute shrinkage and selection operator
ssGSEA: Single-sample gene-set enrichment analysis
DCA: Decision curve analysis
PCA: Principal component analysis
ANOVA: Analysis of Variance
ROC: Receiver operating characteristic
AUC: Area under curve
CNV: Copy number variation
KEGG: Kyoto Encyclopedia of Genes and Genomes
GO: Gene Ontology
RMS: Root Mean Square
GSCs: Glioblastoma stem cells
TMB: Tumor mutational burden
TME: Tumor miccroenvironment
WHO: World Health Organization
IDH: Isocitrate dehy drogenase
