# Supplementary figures and images for: A bioinformatic analysis study of m7G regulator-mediated methylation modification patterns and tumor microenvironment infiltration in glioblastoma
Source: BMC Cancer. 2022 Jul 4;22:729. doi: 10.1186/s12885-022-09791-y (PMC9251941; doi:10.1186/s12885-022-09791-y)

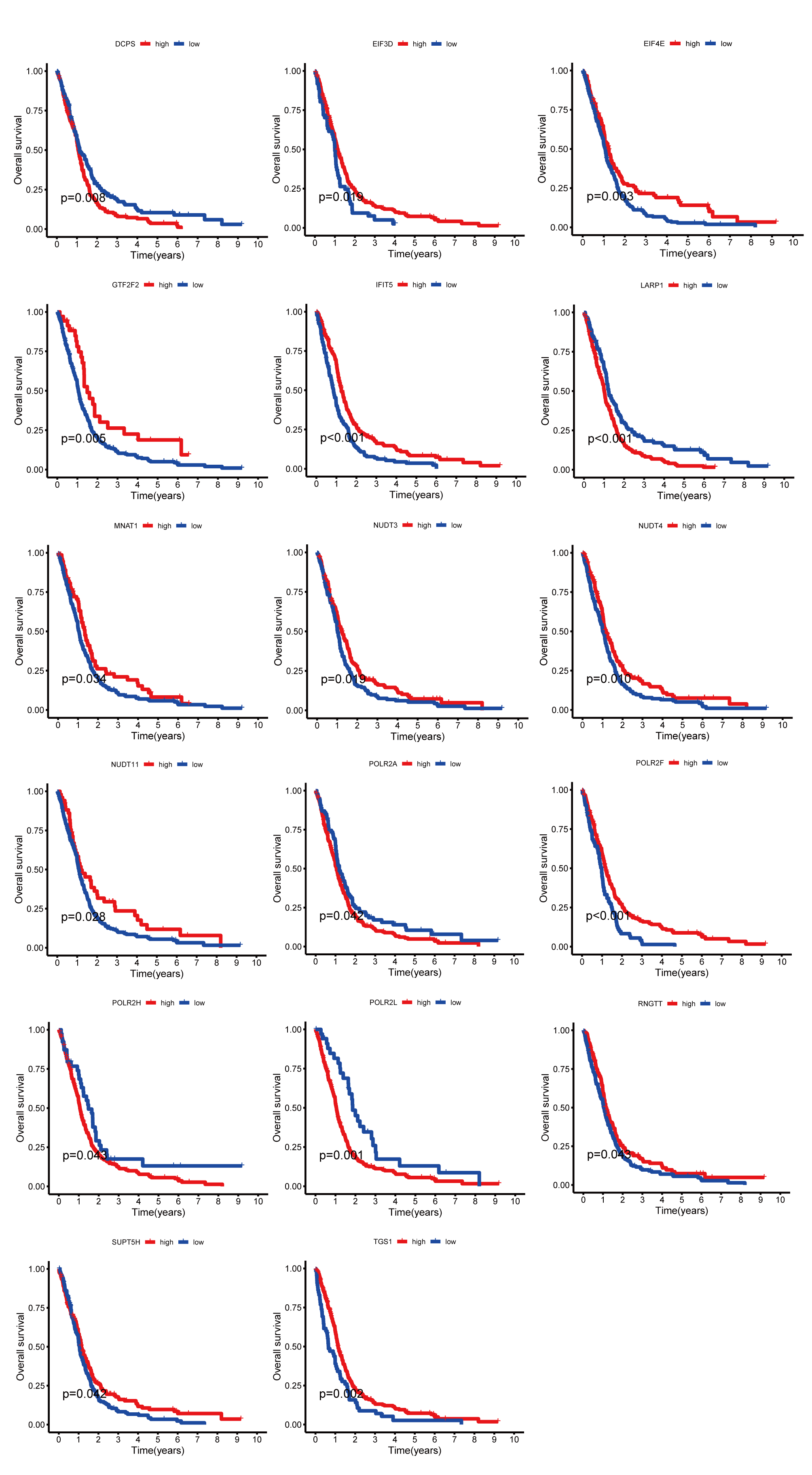

Supplement: Supplementary file 7 — Additional file 7. [file 12885_2022_9791_MOESM7_ESM.tif]

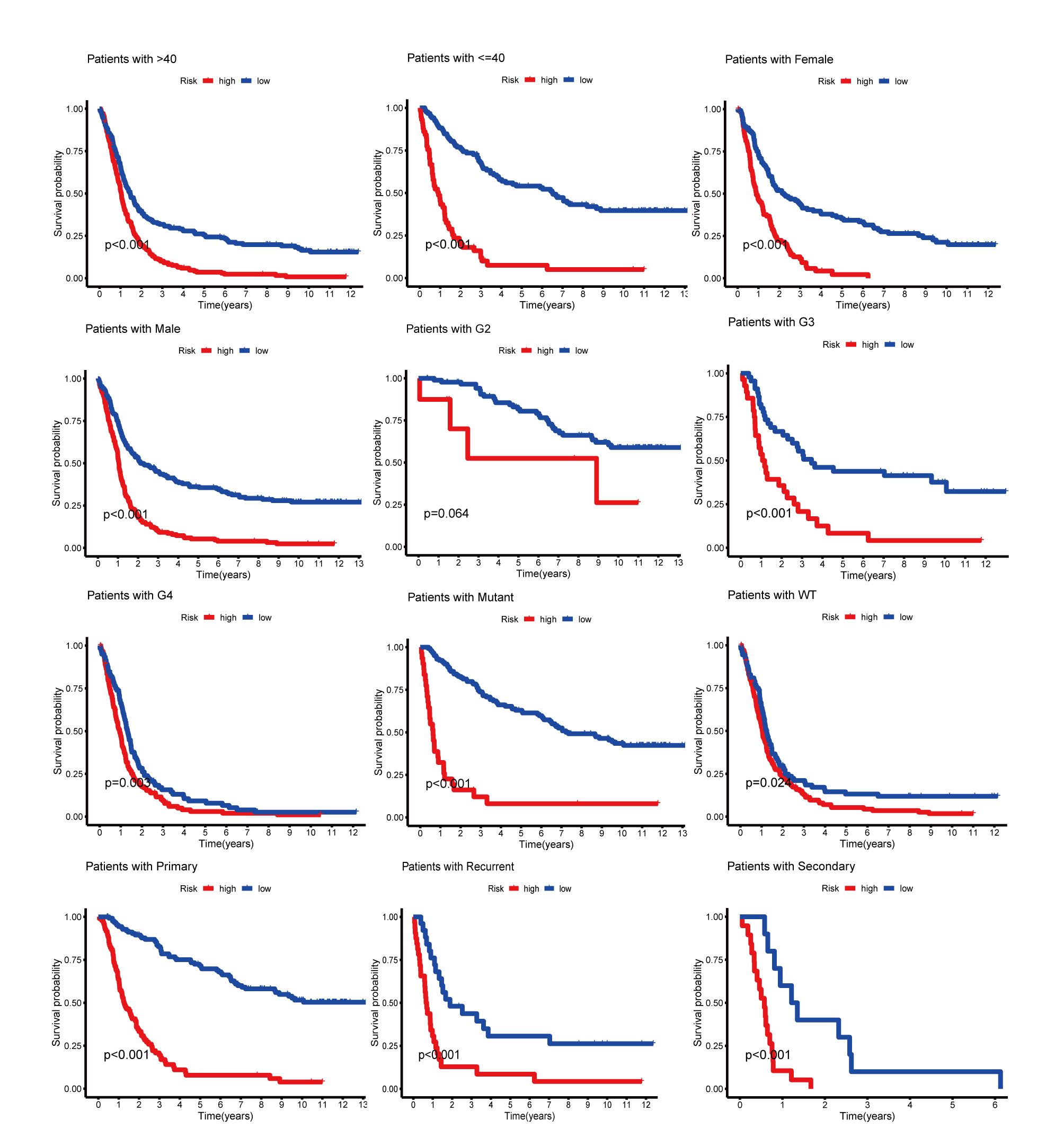

Supplement: Supplementary file 8 — Additional file 8. [file 12885_2022_9791_MOESM8_ESM.tif]

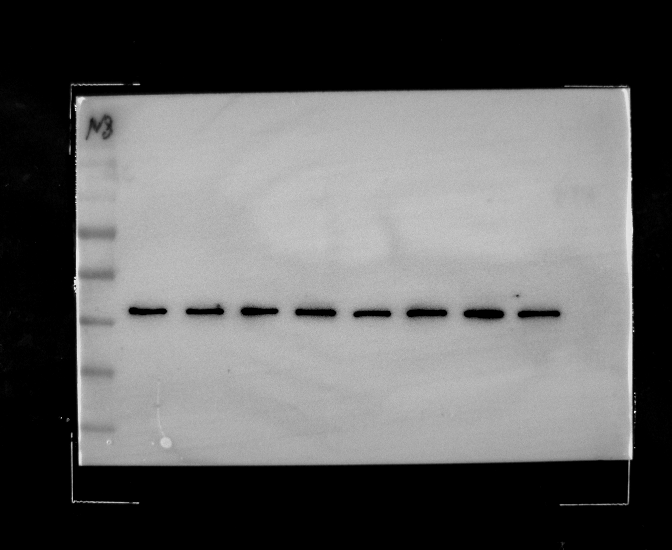

Supplement: Supplementary file 9 — Additional file 9. [file 12885_2022_9791_MOESM9_ESM.tif]

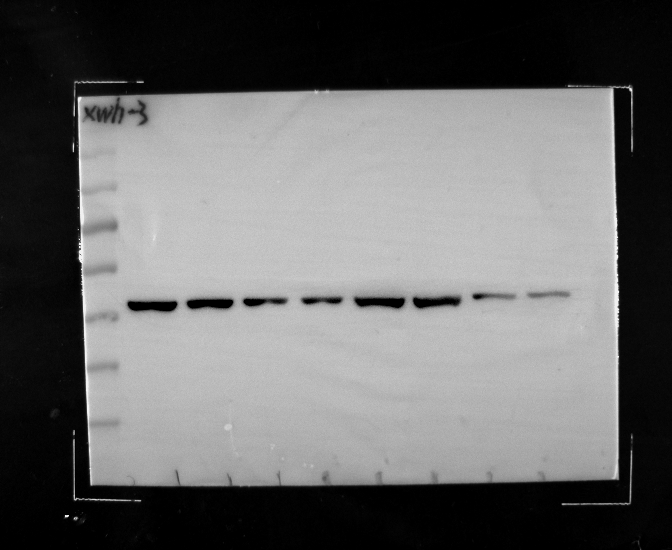

Supplement: Supplementary file 10 — Additional file 10. [file 12885_2022_9791_MOESM10_ESM.tif]
